# Serum ferritin levels and bone mineral density in the elderly

**DOI:** 10.22088/cjim.9.3.232

**Published:** 2018

**Authors:** Mansour Babaei, Ali Bijani, Parnaz Heidari, Seyyed Reza Hosseini, Behzad Heidari

**Affiliations:** 1 Mobility Impairment Research Center, Babol University of Medical Sciences, Babol, Iran; 2 Department of Internal Medicine, Division of Rheumatology, Ayatollah Rouhani Hospital, Babol University of Medical Sciences, Babol, Iran.; 3 Clinical Research Development Unit of Rouhani Hospital, Babol University of Medical Sciences, Babol, Iran; 4 Infectious Diseases and Tropical Medicine Research Center, Health Research Institute, Babol University of Medical Sciences, Babol, Iran; 5 Faculty of Medicine, Tehran Islamic Azad University, Tehran, Iran; 6 Social Determinants of Health Research Center, Health Research Institute, Babol University of Medical Sciences, Babol, Iran

**Keywords:** Association, Bone mineral density, Elderly people, Serum ferritin

## Abstract

**Background:**

Iron overload influences negatively on bone mineral density (BMD) but the results of studies regarding serum ferritin (SF) and BMD are conflicting.This study aimed to determine the association of SF and BMD in the elderly.

**Methods:**

All participants of the Amirkola cohort selected between 2011-2012, aged > 60 years were classified as high or normal (<200ng/ml) SF. BMD at femoral neck and lumbar spine was determined by dual energy x-ray absorptiometry (DXA) and the results were expressed as BMD g/cm2 and BMDT-score. Multiple logistic regression analysis with calculation of odds ratio (OR) and 95% confidence interval was used to estimate the association of low BMD (LBMD) defined as BMD T-score < -1 with SF.

**Results:**

1089 subjects (women, 44.7%) were studied. High SF was observed in 366 (33.6%) and LBMD in 874 (80.2%) subjects. The two groups of SF were similar regarding biochemical parameters and demographic characteristics except MetS, overweight /obesity and diabetes which were more prevalent in high SFgroup. BMD g/cm2 at both measurement sites was significantly higher (P=0.001 for both) and the prevalence of LBMD was significantly lower (74.1% vs 83.1%, P=0.001) in high SF group by OR= 0.60 (0.44-0.81). After adjustment for all biochemical and demographic variables, the association remained significant by adjusted OR= 0. 68 (0.49-0.94).

**Conclusions:**

These findings show a negative association between high SF and LBMD indicating a beneficial effect of high SF in the elderly. Regarding detrimental effect of iron overload on bone mass, these findings require further studies.

Osteoporosis is prevalent in the elderly and in postmenopausal women is an important cause of bone fracture and morbidity  (1). Many factors such as age, sex, hormonal and metabolic disorders, physical inactivities, and smoking are associated with bone loss and low bone mineral density (BMD) (-). However, in the elderly subjects, coexistent of several chronic medical conditions may provide further susceptibility to bone loss. Serum ferritin (SF) which is a marker of iron stores increases by age in both men and women, and in healthy state, its levels ranges from 40-200 ng/ml. On the other hand, SF is also a marker of inflammation which may increase in patients with coexistent chronic systemic inflammatory diseases (6). An association between excess iron and osteoporosis as well as fracture has been shown which is attributed to detrimental effect of iron on bone (7-9). Iron overload decreases osteoplastic activity and facilitates osteoplastic differentiation and bone resorption, furthermore, iron inhibits differentiation of bone marrow mesenchymal cells and bone remodelling, through induction of ferritin (9). In postmenopausal women, excess iron independently affects bone metabolism and leads to low BMD (8).

High level of SF and low BMD are observed in a number of chronic hematological diseases with iron overload such as thalassemia or sickle cell disease  (10, 11), nonetheless, iron deposition does not explain low BMD in all cases of thalassemia (10) and in patients with hematopoietic disorders, low BMD, may be related to not only SF,but also to increased cellularity of bone marrow  (11). The relationship between SF and BMD has been addressed in a few studies with conflicting results (4, 7, -). In a study on the data from the 2008-2010 Korean National Health and Nutrition Examination Survey, comprised 7300 pre and postmenopausal women, Chon et al. found a negative correlation between SF and low BMD only in the lumbar spine (LS) BMD but not in the femoral neck (FN)BMD in premenopausal women (7). Lee et al. in a study of 2,943 subjects aged 65 years and over, found a significantly positive association between high SF and BMD at both FN and LS measurement sites only in men but not in women (13). 

These observations indicate variations in the relationship between SF and BMD according to age, sex or BMD measurement sites. Furthermore, the causes of change in serum ferritin are also important. Since SF as an acute phase reactant, may change in response to inflammatory process and increases in subjects with chronic inflammatory diseases such as obesity, diabetes, chronic obstructive pulmonary disease (COPD), (6, 16). 

These conditions are prevalent in the general population as well as in the elderly people (6, 16). Thus, not only the SF but also the metabolic component of these entities may affect BMD .This context is less studied and data in this field are scarce. We therefore performed the present study to determine the association between SF and BMD in a cohort of elderly men and women comprised all inhabitants of a small town. 

## Methods

The data of this study extracted from the phase 1 of the Amirkola Health and Aging Project (AHAP) cohort study. This longitudinal study cohort study started in 2011 and data collection is repeated every five years (17, 18). All inhabitants of Amirkola, Babol, Iran aged 60 years and over were invited to participate in this project and finally 72.3% of the invited subjects completed the study. All eligible participants entered the study except subjects taking iron containing drug transfusion, antiosteoporotic therapy, chronic systemic or localized inflammatory diseases, current thyroid diseases, hormonal replacement therapy and subjects with impairment in daily physical activities. SF was assessed by standard method and the participants were classified as high or normal SF group according to SF values > 200 or < 200 ng/ml respectively. BMD in the FN and LS was determined by dual energy x-ray absorptiometry (DXA) method using Lexxuss densitometer, and the results were expressed as BMD g/cm^2^, BMD T-score and BMD Z-score. Furthermore, data were provided for demographic features like age, sex, level of education, physical activity, quadriceps muscle strength, frequency of common medical conditions such as obesity, diabetes, metabolic syndrome (MetS), chronic lung and kidney diseases and anemia. 

Biochemical parameters such as serum iron, total iron binding capacity, serum calcium, serum 25-hydroxyvitamin D (25-OHD) were also determined. Details of patient selection, data collection regarding demographic features and biochemical tests have been described elsewhere (19). In brief information with regard to demographic characteristics, physical activity, and medication was provided by interview, review of medical records and fill-in questionnaire. Sample size was estimated to detect a 10% difference in the prevalence of low BMD (LBMD) between subjects of the two groups of high and normal SF. LBMD was defined as BMD T-score < - 1 at either FN or LS. Based on an expected 50% prevalence of LBMD (3), a sample of 388 patients for each group would yield a power of 80 % and confidence interval of 95%. However, we included all participants to compensate the missed data as well as to increase power of the study.

In statistical analysis, the two groups of patients with normal and high SF were compared regarding prevalence of LBMD, FN-BMD g/cm^2^, LS-BMD g/cm^2^. The association of SF with LBMD was determined using chi square test with calculation of odds ratio (OR) and 95% confidence interval (95% CI). Independent association between SF and LBMD was determined using multiple logistic regression analysis with LBMD as dependent and SF as independent variable after adjusting for all covariates. A p-value less than 0.05 was considered significant. SPSS 21 was used to analyze the data. Informed consent was obtained from all the participants. The proposal of this study was approved by the Ethics Committee of Babol Univirsity of Medical Sciences, Babol, Iran

## Results

A total of 1089 patients (487 women with mean age of 67.8+6.8 years and 602 men with mean age of 68.7+6.6 years (P=0.027) entered the study. High SF was observed in 366 (33.6%) patients (females, 47.6%) and LBMD was seen in 847(80.2%) patients. Characteristics of patients with high and normal SF are shown in table 1. In the whole number of patients, high and normal SF groups were similar regarding mean age, physical activity, muscle strength, hemoglobin concentration, serum calcium, serum 25-OHD concentration, level of education and proportion of anemia (table 1). But, the proportion of MetS, overweight/obesity and diabetes was significantly higher in high SF group. Furthermore, serum iron concentrations and BMI were higher and TIBC was lower in high SF as compared with normal SF group. The BMD g/cm2 in both FN and LS measurement sites was significantly higher in high SF (P=0.001 for both) as compared to normal SF group (table 1).

**Table1:** Characteristics of the study population according to serum ferritin (SF) levels

**Variables**	**High SF** **(> 200 ng/ml)** **(n=366)**	**Normal SF(** **<** **200ng/ml)** **(n=723)**	**P values** &
Age (year) (mean±SD)	68 +6.8	68.7+7.1	0.17
Female,no (%)	156(47.6)	331(45.8)	0.17
BMI (kg/m^2^) mean±SD)	27.8+4.3	26.9+4.6	0.002
Overweight/obesity £	265 (67)	451 (63.2)	0.001
Metabolic syndrome, no (%) €	292 (79.8)	507 (70.1)	0.001
Diabetes, no (%)	158 (43.6)	181 (25.1)	0.001
Physical activity score^,^@	11+65	11o.6+62.6	0.15
Quadriceps muscle strength (kg), mean±SD	23.5+10.5	22.5+10.5	0.16
FNBMD (g/cm2), mean±SD₩	0.865+0.164	0.833+0.150	0.0013
LSBMD (g/cm2), mean±SD¥	0.888+0.189	0.855+0.185	0.0059
Serum iron (mc/ml)	91.6+34.1	81.9+35.3	0.001
Serum TIBC * (mc/ml)	277.4+38.5	284.7+38.6	0.004
Hemoglobin (gr/dl)	13.8+1.6	13.7+1.4	0.30
Anemja$, no (%)	65(17.6)	117(16.2)	0.28
Low BMD#, no (%)	273(74.6)	601(83.1)	0.001
Vitamin D (ng/ml) (mean±SD)	32.6+28	35.2+34.1	0.12
Calcium (mg/dl) (mean±SD)	9.2+4.4	9.2+4.3	0.17
Level of education, no(%) Illiterate Primary and high school College	238(65) 96(26.2) 32(8.7)	429(59.3) 238(71.3) 56(7.7)	0.077 0.074 0.25

& Analysis was performed using t-test or chi square test

☆ Quadriceps muscle strength

£ < 25 Normal weight 25-29.=overweight, > 30 Obesity

* Total Iron binding capacity

$ Defined as hemoglobin < 13 gr/dl in men and > 12 gr/dl in women

¥ Lumbar spine BMD

# Defined as BMD T-score < - 1

@ Determined by PAS questionnaire

€ Diagnosed based on ATP III criteria

₩ Femoral neck BMD

Subgroup analysis according to sex demonstrated a significant association between high SF and diabetes as well as serum iron in both men and women. whereas, in men, high SF was also associated with MetS and obesity/ overweight (table 2). The prevalence of LBMD was significantly lower in high SF group (74.6% vs 83.1%, OR= 0.60, 95% CI, 0.44-0.81, P=0.001). After adjusting for all covariates (table 1), the negative association between high SF and LBMD remained at significant level with adjusted OR=0.68, 95% CI, 0.49-0.94, P=0.001) (table 3). Furthermore, there was a significantly negative association of LBMD with MetS, and a notably positive association between LBMD and female sex (table 3). As compared with age group of 60-65 years, there was remarkably positive association between LBMD and aging with a dose- response pattern of relationship as well.

**Table 2:** Association between serum ferritin (SF) and low bone mineral density (LBMD) in the elderly subjects aged 60 years and more after adjustment for other aassociated factors with the calculation of adjusted odds ratio and 95% confidence interval (95% CI)

**Variables**	**Adjusted OR(95%CI)**	**Pvalue**
SF > 200 vs <200 ng/ml	0.68 (0.49-0.94)	0.001
Metabolic syndrome ¥ presence vs absence	0.42(0.28-0.63)	0.001
Females vs males	4.04(2.8-5.8)	0.001
Age groups		
60-64	1	-
65-69	1.04(0.70-1.53)	0.83
70-74	1.78(1.10-2.8)	0.018
75-79	2.85(1.59-5.11)	0.001
80-84	4.17(1.60-10.8)	0.003
> 85	3.61(0.8-16.1)	0.093

Statistical analysis was done using multiple logistic regression analysis.

¥ Diagnosed by ATP III criteria

**Table 3:** Associated factors of high serum ferritin in the study population according to sex

	**Men**			**Women**		
	**> 200** **N=210**	**<** ** 200** **N=392**	**p value**	**> 200** **N=156**	**<** ** 200** **N=331**	**pvalue**
Age (year) (mean±SD)	68.1±6.7	69.4±7.4	0.034	67.9±7	67.8±6.6	0.87
Overweight/obesity £	149(70.9)	208(53)	0.001	116(74.3)	243(73.4)	0.88
Metabolic Syndrome, no (%) €	149(71)	211(53.8)	0.001	143(91.7)	296(89.4)	0.44
Diabetes, no (%)	70(37.8)	84(21.5)	0.02	79(51.6)	97(29.4)	0.0027
Vitamin D (ng/ml)	31.3±25.9	32. 9±30.5	0.49	34.3±30.7	38.6(37.8)	0.18
Calcium (mg/ml)	9.2±0.41	9.2±0.45	0.10	9.2±0.46	9.2 ± 0.42	0.10
Physical activity score (mean±SD) @	113.8±71.2	105±68.6	0.13	120±57.6	116±54.1	0.45
Muscle strength (kg) ☆	29±9.5	28.1±10	0.20	15.9±6.3	15.9±6.5	0.10
Serum iron (mean±SD)	94.3±34.5	86.6±37.4	0.013	87.8 ±33.2	76.2±31.6	0.001
TIBC (mean±SD) &	276.7±7 38	282±39	0.10	278 ± 39.2	287±37.8	0.10
Hemoglobin gr/dl) (mean±SD)	13±1.5	13±1.4	0.10	13 ±1.54	13±1.2	0.10
Anemia, no (%) $	34(16.2)	57(14.5)	0.82	31(19.9)	60(18.1)	0.83
FN-BMD (g/cm2) (mean±SD) ₩	0.918±0.156	0.875±0.146	0.001	0.755±0.148	0.784±0.140	0.036
LS-BMD (g/cm2) (mean±SD) ¥	0.955±0.179	0.921±0.180	0.027	0.799±0.163	0.777±0.159	0.15
LBMD, no (%) #	140(66.6)	301(76.7)	0.025	133(85.2)	300(90.6)	0.098
Education levels, no (%) Illiterate Primary and secondary school High school & College	128(61) 51(24.3) 31(14.8)	226(57.6) 119(30.3) 47(12)	0.53 0.42 0.72	110(70.5) 45(28.8) 1(0.6)	203(61.3) 119(36) 9(2.7)	0.10 0.38 0.89

& Analysis was performed using t-test or chi square test

£ < 25 Normal weight 25-29.=overweight, > 30 Obesity

€ Diagnosed based on ATP III criteria

& Total Iron binding capacity

₩ Femoral neck BMD

¥ Lumbar spine BMD

@ Determined by PAS questionnaire

☆ Quadriceps muscle strength kg

$ Defined as hemoglobin < 13 gr/dl in men and > 12 gr/dl in women

# Defined as BMD T-score < - 1

## Discussion

The findings of this study indicate that high SF in the elderly people aged 60 years and more is associated with lower prevalence of LBMD at both femoral neck and lumbar spine. There was also a positive dose-response pattern of association between LBMD and age, as well as a negative association between MetS and LBMD. These findings are consistent with the results of earlier studies (4,13,20). A positive association of high SF and BMD in all B7MD measurement sites has been reported in the elderly men aged > 65 years (13). Another study found an independent positive association between BMD and serum ferritin in postmenopausal women (4). In a retrospective analysis of postmenopausal women without hematological disorders aged 60-83 years, Mousal et al. found a positive association between SF and BMD at both FN and LS (20).

Nonetheless, a number of studies found a negative association between SF and BMD. Chon et al. discovered a trend toward decreased LS-BMD only in premenopausal but not in postmenopausal women, whereas there was no association between SF and FNBMD (7). Similarly, Kim et al. in a population based cross-sectional study found an inverse association of SF with BMD at all measurement sites only in women > 45 years of age and an increased odds of prevalent osteoporosis and fractures in high SF quartiles (21). A similar negative association between SF with LSBMD and FNBMD has been reported in women aged > 48 years (14). In a study of women with hip fractures, SF > 150 ng/ml was associated with L BMD and SF was negatively correlated with both FN-BMD and LSBMD and positively with bone turnover markers (18). 

A 3-year retrospective longitudinal study of the elderly men and women showed a relationship between SF and BMD loss. In addition, there was a dose- response association between SF and the rate of BMD loss at all proximal femur regions in both men and women. The authors found iron stores as an independent risk factor of accelerated bone loss even in healthy women. A negative correlation of SF and BMD changes has been observed in patients with hematopoietic diseases with abnormal proliferation of bone marrow cells  (11).

Variations in the relationship between SF and BMD across different studies may be attributed to factors such as, study design, age, sex, and site of BMD measurement. Nevertheless, coexistence of disease conditions especially chronic lung and renal diseases, diabetes, MetS, and obesity which are prevalent in the general population as well as in elderly patients can confound the results (12, 22-24). These conditions are usually associated with inflammatory process and thus, may impress on both SF and BMD sera (6, 25). In the present study, high SF was significantly associated with MetS, diabetes and overweight/obesity. All of these conditions are associated with high BMD and increased level of SF (4, 5, 26-28). 

Although, the significant negative association between SF and LBMD in the present study remained statistically significant after adjustment of these conditions, but coexistence of obesity in all mentioned conditions justifies a possible confounding effect of growth hormone/insulin growth factor-1 axis (29). These observations suggest that, studies which address the issue of SF and BMD in the elderly should consider the confounding effects of obesity-related factors which can affect on both BMD and SF (26-32). The results of studies have limitations regarding study design in which the association does not indicate causality. In statistical analysis, we did not compare men and women separately. The prevalence and the associated factors of osteoporosis in men are different from women (4, 5), yet, the results would not be affected, because distributions of these factors are expected to be similar across the two comparison groups. Lack of data regarding the cause of high serum ferritin is another limitation. The strength of this study is dependent on patient selection which comprised all the inhabitants of a town. Another strength is homogeneity of the study population regarding ethnicity, lifestyle, and other associated factors of bone loss. 

In conclusion, the findings of this study indicate a positive association between SF and BMD in the elderly people aged 60 years and older. Regarding the negative effect of iron overload on bone mass and contradicting results across relevant studies, this context needs further investigations with emphasis on age, gender of the study population, BMD measurement sites and in particular coexistent chronic medical diseases in the older subjects. Many chronic coexisting diseases in the elderly affect SF and BMD differently and so may result in unexpected findings.
